# Breast Cancer and Its Impact on Individual Personality Through a Dimensional Perspective: A Literature Review

**DOI:** 10.7759/cureus.64348

**Published:** 2024-07-11

**Authors:** Oltean Andra, Andrei Manea, Aurel Nirestean, Raluca Niculescu, Strete Elena-Gabriela

**Affiliations:** 1 Department of Psychiatry, Emergency Clinical County Hospital of Targu Mures, Targu Mures, ROU; 2 Department of Radiology, Emergency Clinical County Hospital of Targu Mures, Targu Mures, ROU; 3 Department of Psychiatry, George Emil Palade University of Medicine, Pharmacy, Science, and Technology of Targu Mures, Targu Mures, ROU; 4 Department of Pathophysiology, George Emil Palade University of Medicine, Pharmacy, Science, and Technology of Targu Mures, Targu Mures, ROU

**Keywords:** holistic intervention, personality disorders, anxiety, depression, breast cancer

## Abstract

Psychiatric pathology stands out in contemporary society not only as independent but also through its association with other medical comorbidities such as neoplastic diseases. Specialized literature confirms over time the coexistence of these diseases. There is a tendency to develop various psychiatric manifestations such as mood disorders and somatoform disorders, as well as decompensation of underlying existing psychiatric pathologies (anxiety disorders and psychotic disorders) or personality disorders (a good example is the exacerbation of anxiety in obsessive-compulsive personality disorder). Breast cancer, like any disabling disease, affects the person's psyche and behaviors as a whole. It is scientifically proven that mental balance influences the quality of life of patients and also the evolution and prognosis of the disease, psychological processes being able to modulate the activity of the tumor process. It is necessary to expand clinical practice and research beyond the simple evaluation of symptoms, and the goal of treatment should not only be to reduce symptoms but also to improve in terms of both physically and mentally the quality of life of cancer patients.

## Introduction and background

Breast cancer (BC) is the most prevalent noncutaneous malignancy diagnosed in women in the Western World, with one in every eight women having it during her lifetime [1]. Faced with BC, the patient is subjected to new and tough problems and decisions, which adds to his or her stress. Accepting a diagnosis, going through treatments, understanding the prognosis, dealing with possible side effects, managing a potential relapse, and facing an unknown future are all components of a stressful process. This process can cause psychological instability and lead to depression or other mood disorders.

Psychiatric pathology is of a particular diversity due to the comorbid organic conditions that are often associated with it. They interfere with the dynamics of mental illness and episodes and also with personality traits and dimensions. Thus, the onset, evolution, prognosis, and therapeutic responsiveness of psychopathological diversities can also have somatic conditioning. Also, clinical practice always confirms the mutual inter-conditioning between personality traits and dimensions and various somatic diseases [2-5].

Any change in health status, regardless of etiology, is considered to be a bio-psychological and socio-cultural impasse. Suffering is perceived on a psychic level and represents a complex of states, sensations, perceptions, reasonings, assumptions, and representations, all under the sign of hope and despair [6].

Mental health is generally defined by awareness capacity, self-concept acceptance, mastery of the environment and the ability to cope with daily requirements, integration and unity of personality, self-confidence and autonomy, realistic perception of the society in which a person lives, openness to new experiences, and personal development [7]. A healthy person presents a unitary personality structure, in which all complementary components function integratedly, not disruptively, is aware of their own limits, and can cope with them [7].

Personality traits are directly involved both in favoring and triggering episodes and mental illnesses as well as in the diversity of evolutionary and therapeutic response modalities. They also have a major role in subsequent psycho-social rehabilitation. Personality factors can impact behavioral changes after a diagnosis. Individuals with proactive personalities, for example, may participate in health-promoting activities and seek information more frequently [8].

The objective of the narrative review is to confirm or deny the direct relationships between the clinical-evolutionary particularities of BC and individual personality traits.

## Review

In the assessment of personality, two major directions are distinguished: the identification of specific traits that allow the formulation of a categorical diagnosis where it can be outlined as well as the assessment of the person's traits through the prism of personality dimensions and in the absence of a categorical framework.

Personality disorders can be diagnosed from a categorical point of view

**Figure 1:**
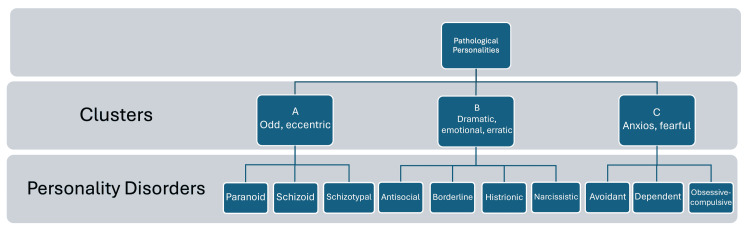
Pathological personalities divided into clusters. Source: American Psychiatric Association 1994.

The diagnosis of a personality disorder is confirmed by the presence of a certain number of specific criteria established by consensus (DSM IV-R). Thus, from a categorical perspective, 10 pathological personalities divided into three clusters, A, B, and C, are diagnosed, as can be seen in Figure 1. These include, in order, schizoid personality disorder, schizotypal personality disorder, paranoid personality disorder, narcissistic personality disorder, antisocial personality disorder, borderline, histrionic, avoidant, dependent, and obsessive personality disorder [8].

The DSM diagnostic system has gone through several major changes from the time of its first publication to the latest edition. Most changes are related to the addition or even removal of diagnoses or criteria [8,9].

Despite the attempts of the Operative Group for personality disorders in DSM-V to significantly modify the 10 diagnoses of personality disorders, they still remain unchanged, being described according to the same diagnostic criteria from DSM-IV-TR in Part II of DSM-V [8].

Part III instead includes the Operative Group’s new Alternative Model for Personality Disorders where only 6 of the preexisting 10 are listed: avoidant, borderline, narcissistic, obsessive-compulsive, and schizotypal personality disorder [8]. 

With the help of this dimensional model, we can diagnose a personality disorder based on traits. These traits derive from the Big Five model and personality psychopathology [8]. Trait domains are negative affectivity versus emotional stability, detachment versus extraversion, conflictuality versus agreeableness, disinhibition versus conscientiousness, and psychoticism versus lucidity [9].

According to the DSM-V, pathological personalities are considered to be abnormalities of the ego, that is, of consciousness, self-esteem, self-control, self-evaluation, emotion regulation, and self-direction capacities [2]. The adaptive deficit of certain types of personalities is independent of conjunctural factors but the latter can also activate maladaptive behavior [8]. Both normal personality traits and disharmonious traits are unconscious or partially conscious and are also stable over time. Lenzenweger (1999) and Cloninger et al. (1993) describe the fact that genes are responsible for persistent personality traits [10,11].

The impact of the diagnosis of BC can produce certain irreversible damage to the psyche when we relate to disharmonious personalities. Therefore, the psychiatric examination of patients diagnosed with BC is mandatory from the beginning, thus being able to prevent the occurrence of psychopathological episodes and to start early treatment when needed [12]. Optimistic patients showed better coping strategies, such as problem-solving and seeking social support, which were associated with improved survival rates. Conversely, patients with higher neuroticism had poorer coping mechanisms, such as avoidance and denial, leading to worse outcomes.

From a somatic point of view, there are differences in the evolution of diseases, but the way in which they are perceived and experienced largely depends on the dimensions of the personality (for example, an anxious personality style will see it as "catastrophizing") [13].

In pathologies that have a surgical solution, where the suffering manifests itself shockingly and on a psychological level, the surgical procedure must always be completed with the intervention of a psychotherapist or psychiatrist. A series of special means should be used, aimed to reduce the anxiety or depression of the patients generated by the disease and amplified by the perception of operative risk. Psychotherapy, used in surgical patients with major psychological problems, has an increasingly important role in the success of the operative act [14].

In specialized literature, the issue of psychotherapy and the impact of a serious illness such as BC on the personality of a patient is very widespread, especially lately. Thus, although there are numerous theoretical studies that manage to capture very well the dynamics of these concepts taken individually, there are few attempts to approach the correlation between BC, the personality changes of the woman diagnosed with this disease, and the psychotherapy applied to this person [15,16].

Breast cancer - a major global health problem 

At the beginning of the 21st century, cancer was and continues to be a major global health problem, representing the second cause of death after cardiovascular diseases. From the point of view of incidence, within oncological pathology, the highest frequency at the European level is represented by BC (464,000 newly diagnosed cases annually). Cancer affects one out of two people throughout their lives and the risk of occurrence gradually increases with age. The International Agency for Research on Cancer estimates a substantial increase in the number of patients with newly diagnosed neoplastic pathology by the year 2025, 19.3 million annually [17].

In today's Romania, BC is taking on a worrying scale. More than 12,000 new BC patients are diagnosed each year, making it the second leading cause of cancer-related fatalities, after lung cancer [18]. And yet, a lot of medical and psychological problems related to the appearance, evolution, and therapy of the disease have not been elucidated [19,20]. Although the survival rate of patients diagnosed with BC has improved among the countries of the European Union in the last decade (from 79% to 83%), Romania continues to be in the last position with 75% survival at five years, above Lithuania (74%) [21].

Like any disabling disease, BC affects the overall psyche of the person and the personality traits. This is because a chronic evolution is always confirmed, various complications appear, and the image and self-esteem are always affected - in variable proportion - which is associated with the slippage of the level of involvement and deficiency in all the roles of life and in interpersonal relationships [20,22,23]. Although the importance of psychological support in cancer patients is widely accepted, universal standards for psycho-oncological care have not yet been established [24]. In Romania, psychological intervention has not only not entered current practice but is also not accepted by the entire mass of population. Considering the imperative need for psycho-emotional support felt by the parties involved in cancer treatment, oncology medicine in collaboration with psychiatry must contribute to the development of the integration of the concepts of psycho-oncological (holistic) care [24].

The absence of the breast, considered a symbol of female beauty, represents serious damage to one's image, which can generate important psychological and adjustment disorders, equally altering one's self-image, family, social, and professional life. The attitude of patients toward BC is different depending on the mental balance of each one and, of course, depending on the personality, this hypercomplex system subordinate to the social and cultural environment, made up of all the traits of temperament, character, intellect, and the volitional energetic ones [25,26].

Cancer is a complex disease that involves a rapid and uncontrollable multiplication of cells and is associated with deep distress and anguish. Because the terms "emotional," "psychological," or "psychiatric" continue to be associated with stigma, the NCCN (National Comprehensive Cancer Network) chose the term distress, which is a more appropriate and less stigmatizing concept. According to the same organization, 20-40% of people diagnosed with BC experience significant distress, yet only about half seek treatment from a psychiatrist or psychologist, despite the fact that their mental state requires it. Among them, a significant impairment in the level of quality of life and lower therapeutic compliance are noted [6].

Internationally, in the 90s following Holland's integrative model of adaptation to the stress of neoplastic disease, five emotional problems present in BC patients were identified: the emotional charge of the word cancer, lack of control over one's own existence perceived by the patient, the uncertainty regarding the evolution of the disease, the emotional problems created by the treatment itself, and the debilitating effect of applied therapies [27].

Modern research and clinical practice point to the fact that half of the BC patients have different psychiatric or psychological disorders, which need optimal diagnostics and therapy [28]. The depressive effect is considered to be normal, which is a result of the insight into the permanent inability to adapt to the environment [29].

A study carried out in 2017, in America, on a group of 1248 patients diagnosed with various types of cancer, including BC, highlights the fact that following the application of personality assessment questionnaires, people with a high level of distress have high neuroticism and low extroversion, agreeableness, and conscientiousness [30].

In his well-known work "The Death of Ivan Ilyich," Tolstoy describes the consequences generated by the family and the doctor, who hid the diagnosis, not taking seriously the intense stomach pain caused, in this case, by gastric cancer. He had to fight alone with fears that he was seriously ill, suffering from an incurable disease, while his family maintained a conspiracy of silence. Social stigma, shame, and fear were and continue to be elements associated with a cancer diagnosis.

One of the most recent studies carried out at the beginning of 2018, Turiano's study, on a group of 55 patients diagnosed with BC confirms the fact that increased conscientiousness and low neuroticism are associated with a higher rate of therapeutic success and acceptance and assumption of the disease [31].

Personality assessment in patients diagnosed with other pathologies outside the psychiatric spectrum, including BC, is deficient at a national level. A cancer diagnosis is perceived by many as a death sentence. The related existential threat initiates substantial suffering, even more so if we are talking about persistent pain, then hopes are reduced, fears fueled, the pain intensifies, and the person feels alone and abandoned [32].

In cancer, we always talk about a process with a double meaning: one biological and one psychological [13,32]. The psychic reaction to these losses is varied, from demoralization to passivity, from memory problems to anger [16]. The biggest psychosocial impact is BC, which is the most common type of cancer among women, due to low self-esteem, increased mortality rate, and finally, the relationship with the environment [24,33,34].

BC is associated in most cases with mental disorders such as depression that coexist with anxiety and pain. It is a real challenge most of the time to study mood changes among BC patients because they vary greatly from sadness to major depression, and these swings are sometimes difficult to manage especially when it comes to patients who have repeatedly faced borderline situations throughout their lives [33,34]. Depression negatively affects the patient's quality of life, physical activity, and interpersonal relationships, and also leads to a decrease in treatment compliance, which is associated with a decrease in the survival rate [35].

Exploration of the relationship between personality traits and mental health has revealed that certain traits may predispose individuals to psychological disorders or, conversely, contribute to overall well-being [30].

It is complex and multifaceted how personality traits and psychiatric disorders are related. For clinicians to provide the most effective and personalized care, they must be aware of the role that personality traits play in the onset, progression, and outcomes of psychiatric disorders. Our knowledge of these connections will continue to be improved by further research in this area, which will also guide best practices in clinical settings [13,33].

Women diagnosed with BC live with the burden of their disease, the treatment, and the psychosocial consequences of the disease, all of which contribute to experiencing severe psychological suffering. These sufferings involve adapting to the status of a cancer patient, the existential dimension of the disease, the relationship with the family, the search for a spiritual or religious belief that gives them moral support, and an explanation of the meaning of life and death [25,26,32]. A therapeutic approach that also considers the dimensions of personality - with its adaptive and maladaptive particularities - can play a significant role in this regard [15]. We can mention here cognitive-behavioral therapies, pattern-centered psychotherapy, mindfulness, and structured treatment interventions [36,37].

## Conclusions

Individual personality traits play a fundamental role in awareness, processing, and interpretation of the severity of a diagnosis such as BC. A multidisciplinary therapeutic approach that also considers the assessment of personality dimensions - with its adaptive and maladaptive particularities - can have a significant role in this regard. The emotional response to a BC diagnosis can vary greatly depending on personality. Some may experience denial, while others might face intense fear or sadness. Most of the time, psychiatric symptoms remain unassessed and undiagnosed, thus reducing the quality of life, shortening survival time, reducing treatment compliance, and prolonging hospitalization. Effective symptom management is associated with improved quality of life, better psychological adjustment, and improved disease understanding, decision-making, treatment adherence, and treatment response.
